# UAV-Driven Structural Crack Detection and Location Determination Using Convolutional Neural Networks

**DOI:** 10.3390/s21082650

**Published:** 2021-04-09

**Authors:** Daegyun Choi, William Bell, Donghoon Kim, Jichul Kim

**Affiliations:** 1 Department of Aerospace Engineering & Engineering Mechanics, University of Cincinnati, Cincinnati, OH 45221, USA; choidg@mail.uc.edu; 2 Dynetics, Huntsville, AL 35806, USA; wjbell@bu.edu; 3 Department of Aerospace Engineering, Mississippi State University, Mississippi State, MS 39759, USA; jc.kim@ae.msstate.edu

**Keywords:** crack detection, deep learning, convolutional neural network, image processing, unmanned aerial vehicle

## Abstract

Structural cracks are a vital feature in evaluating the health of aging structures. Inspectors regularly monitor structures’ health using visual information because early detection of cracks on highly trafficked structures is critical for maintaining the public’s safety. In this work, a framework for detecting cracks along with their locations is proposed. Image data provided by an unmanned aerial vehicle (UAV) is stitched using image processing techniques to overcome limitations in the resolution of cameras. This stitched image is analyzed to identify cracks using a deep learning model that makes judgements regarding the presence of cracks in the image. Moreover, cracks’ locations are determined using data from UAV sensors. To validate the system, cracks forming on an actual building are captured by a UAV, and these images are analyzed to detect and locate cracks. The proposed framework is proven as an effective way to detect cracks and to represent the cracks’ locations.

## 1. Introduction

Preserving structures’ integrity is critical to maintaining their safety. The growing size and complexity of infrastructure have made maintenance a challenge. According to the American Society of Civil Engineers’ (ASCE) Infrastructure Report Card for 2017 [1], 9.1% of 600,000 bridges are structurally deficient in the United States and one in five bridges are at least 50 years old. The ASCE estimates that the backlog for necessary refurbishment has grown to $123 billion. Currently, the Federal Highway Administration requires a bridge to be inspected every two years. These inspections are time-consuming and require trained individuals with specialized equipment to inspect the entire structure. Another difficult challenge for inspectors is inspecting buildings that have been damaged with potential danger to enter. While the exterior of the building may be inspected, the possibility of partial or total collapse may prevent further inspection when they are uncertain of the extent of the damage to the structure’s interior. New technologies can augment inspectors’ abilities to monitor a number of deteriorating bridges, and a system for remotely observing the damage helps inspectors ensure safety. With more automated monitoring systems, the frequency at which structures are monitored would be increased dramatically.

Cracks in structures are typically examined by nondestructive evaluation technologies. A visual inspection using high-resolution photography is the dominant method to inspect structures among several nondestructive technologies, such as impact-echo, ground-penetrating radar, ultrasonic surface waves testing, high-resolution photography, etc. [2,3]. Currently, inspectors must visit sites with a device to acquire visual information. However, unmanned aerial vehicles (UAVs) have been used to acquire images recently because it is easy to capture images in dangerous and/or unreachable places. Previous investigations using machine vision for crack detection have used image processing algorithms, such as Hough transform, wavelet transforms, region-based segmentation, Hessian matrix-based vesselness measurement technique, and principal component analysis to identify patterns in images that indicate cracks [4,5,6]. More recently, machine learning and deep learning algorithms to identify cracks have also become a popular method of crack detection [7,8,9,10]. Convolutional neural networks (CNNs), which are deep learning techniques, excel in object recognition applications because they are excellent at extracting features from large data sets that make adept machine learning models for object recognition [11,12,13]. Furthermore, the increasing affordability of graphics processing units (GPUs) has popularized lowering CNNs’ barrier-to-entry arising from the intense computational cost of training them [14]. For these reasons, there are many previous cases of their use in damage detection applications. In Reference [15], the authors trained CNNs on photometric stereo images of rail surfaces and utilized regularization methods to alleviate overfitting with a small training data set. For crack detection in concrete structures considering varying real-world conditions (e.g., lighting and shadow changes), CNNs were trained using various images and combined with a sliding window technique to scan any image larger than the input resolution [16]. For road crack detection, a CNN model with ConvNet architecture was adopted in Reference [17]. Wang et al. [18] proposed a two-state data augmentation method to improve the quality of crack detection. By applying the rotation method among several data augmentation methods in state one and the greedy algorithm in state two, the model was effectively trained and showed high performance for crack detection in concrete structures. Among various CNN models, the VGG16 model has been widely utilized until now. Wei et al. [19] proposed a fastener defect detection approach on a railway track using CNN, especially the VGG16 model because it can simplify the classification procedure compared to the conventional image processing technique. In addition, Yang et al. [20] utilized the VGG16 model as a base model for their study. They proposed a transfer learning method based on a deep CNN for civil infrastructure crack detection. Qu et al. [21] used the LeNet-5 to classify crack images among disordered input images and optimized the VGG16 model to detect concrete pavement cracks on the images. Moreover, studies using the VGG16 model were performed in a variety of fields, such as combustion (classification individual combustion variants [22]), agriculture (disease classification in eggplant [23]), environmental protection (classification of recyclable garbage [24]), and medical science (classification of brain tumors [25]).

Previous studies [15,16,17,18,19,20,21] focused primarily on increasing the performance of crack detection algorithms. In addition, a number of studies have used cameras installed in UAVs to capture visual data only [26,27,28,29,30]. In this work, a UAV’s position information as well as images taken by the UAV’s camera are utilized. With this information, a proposed framework not only identifies cracks but also provides their location information. Image processing techniques are initially utilized to merge the obtained image into a large image, and a CNN based on the VGG16 model is applied to identify cracks. Then, with the UAV’s position information and outcomes of the deep learning algorithm, cracks’ location information in the world coordinate system is obtained. Lastly, it provides a full crack region map that contains the cracks boxed with red and their location information. To the best of the authors’ knowledge, this work is the first framework that can provide the presence of cracks and their locations in the world coordinate system at the same time. By combining the crack detection capability with the provision of cracks’ locations, the efficiency of large structures’ health monitoring can be improved. This proposed framework provides inspectors with the information they need before setting foot in the building, and this helps to reduce their time-consuming work. Implementation of such a framework would serve to augment inspectors’ abilities to fully inspect large structures for evidence of damage through the detection of cracks on the structures’ surface.

## 2. System Architecture

**Figure 1:**
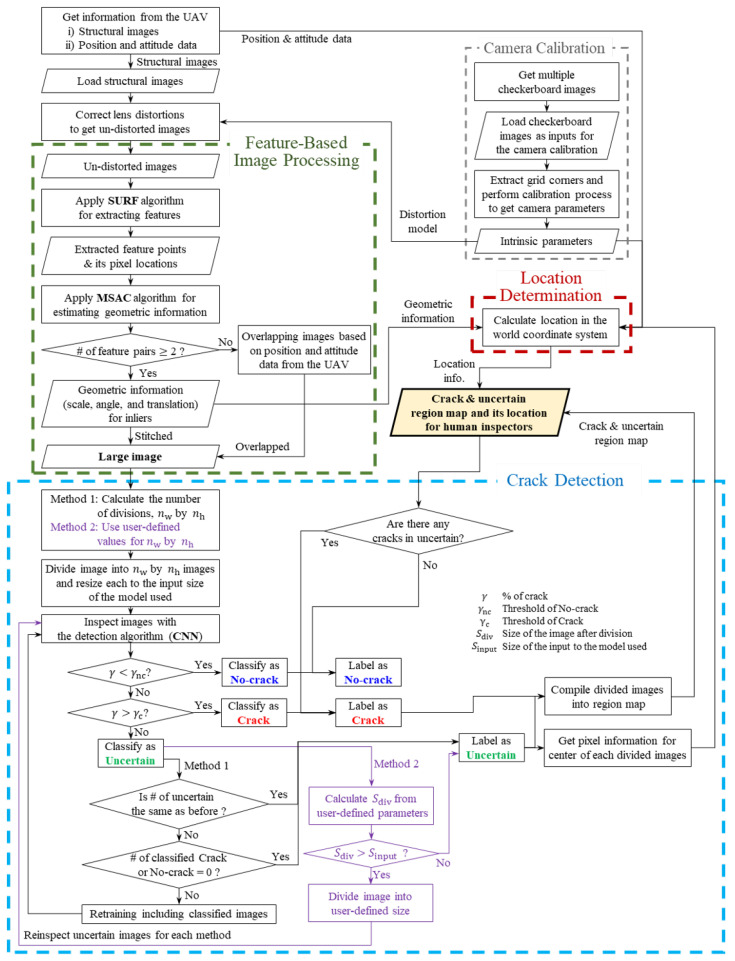
Flowchart of the system proposed.

The framework proposed in this work is largely composed of three parts: (i) feature-based image processing, (ii) crack detection, and (iii) location determination. The flow of the suggested framework is described in Figure 1. First, image processing techniques are used to extract features in images and to merge them into one large image. Additionally, to apply this process, a camera calibration process for extracting parameters used in this process is performed. Next, cracks in the stitched large image are identified by using the deep learning model, and cracks’ pixel coordinates about locations on the image are obtained. Finally, the location in the real world is calculated, and the crack region map, which includes the marked crack part and its location, is provided.

### 2.1. Feature-Based Image Processing

Taking multiple images in different views is the general method of capturing a large structure because of limitations for the field of view and/or the resolution of a camera. It is possible to check the structure’s crack conditions by looking at each image, but it would be difficult to distinguish the location of cracks, especially with similar images. For this reason, the entire image map for the structure would be useful for the health monitoring of the structure; thus, the image stitching process is considered. Before the image stitching process, distortion of the structural images is compensated by using the distortion model obtained by the camera calibration process.

The image stitching algorithm consists of two parts: detecting features and estimating the geometric relationship between images. Scale-Invariant Feature Transform (SIFT) [31] and Speeded Up Robust Features (SURF) [32] are the most popular methods to extract features. SURF is used in this work because of its time efficiency comparing to SIFT [33]. Plenty of features are returned by this algorithm including unreliable features, which are called outliers.

Inliers, which are correct matching features, and geometric relationships between images are obtained by using feature matching methods, such as Random Sample Consensus (RANSAC) [34] and M-estimator Sample Consensus (MSAC) [35]. In this work, MSAC is utilized to handle the outliers and to estimate parameters for homography because MSAC is more robust than RANSAC in terms of the change in the threshold of the cost function expressed as

(1) $$C\;=\;\sum_{i}\rho \left(e_{i}^{2}\right),\;\mathrm{where}\;\rho \left(e_{i}^{2}\right)\;=\;\left\{\begin{array}{cc} e_{i}^{2}, & e_{i}^{2}\;<\;T^{2},\\ T^{2}, & e_{i}^{2}\;\geq \;T^{2}. \end{array}\right.$$

where $\rho (e_{i}^{2})$ is the error function for the geometric distance and *T* is the threshold. Using MSAC, inliers and geometric information are estimated. This geometric information contains scale, rotation, translation, reflection, and shearing between images, but the scale, rotation, and translation are only considered the similarity transformation in this work because reflecting and shearing images are not taken by a UAV. That is, geometric information is used to determine how the images are merged by finding the correct feature matching set for images. For the similarity transformation, it is required to get more than two pairs of feature points.

The applicability of stitching algorithms would be determined by the available images providing sufficient features for matching. If sufficient features are not provided for stitching, an overlapping image using position, distance, and attitude data obtained from a sensor of the UAV is an alternative option. Obviously, the overlapped image may not provide clear information compared to the stitched image because of measurement errors.

### 2.2. Crack Detection Using CNNs

#### 2.2.1. VGG16 Model

**Figure 2:**
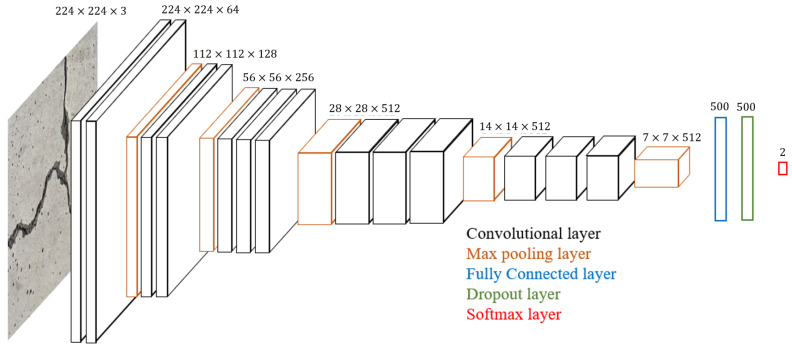
Architecture of the modified VGG16 model.

For crack detection, the VGG16 model is utilized because of its high performance in ImageNet and transfer learning applications. Additionally, it has been used extensively for object recognition in disparate arenas. The architecture of the VGG16 model introduced by Simonyan et al. [36] is visualized in Figure 2. This model supports images with size 224 by 224 and 3 channels and uses 16 convolutional layers with 3 by 3 filters and increasing depth to optimally extract features in the input image. At the end of a stack of filters, a 2 by 2 max-pooling layer is used to reduce the volume of the information. In the ImageNet model, the final max-pooling layer is connected to a single fully connected layer 4096 neurons long, which is then connected to a softmax layer for 1000 classifications. For this work, *the fully connected and softmax layers were removed and replaced with a fully connected layer 1024 neurons long and a softmax layer for 2 classifications* as shown in Figure 2. In this way, the output is essentially a real value between 0 and 1. On inspection, 1 and 0 represent maximum confidence in the presence of a crack and no crack, respectively.

#### 2.2.2. Data Set

The crack detector was trained on a collection of images from a data set retrieved from Mendeley compiled by Maguire et al. [37] called SDNet to support machine learning applications for structural health monitoring. This data set contains 56,000 images of bridge decks, walls, and pavements. To ensure the data set had good quality, the images were manually inspected. From SDNet, good quality images for each category, crack and no crack, were selected: 1800 crack and 1800 no-crack images, respectively. From the pool of 3600 images, 80% of images were stored in the training directory and 20% were stored in the validation directory.

#### 2.2.3. Training

Training of the model consisted of fine-tuning the weights learned in the ImageNet competition. When retraining deep learning models, there are choices in the depth of the retraining. The model can be retrained deeper by increasing the number of convolutional layers to be fine-tuned. Many previous works employing transfer learning [38,39,40] have demonstrated varying performance with varying depths of retraining. Typically, researchers observed increasing performance with increasing depths of training at the cost of computation time. In this work, the model was fine-tuned in its entirety by using a computer that has a GTX 1080 Ti GPU with 11 GB VRAM and 3584 CUDA cores, 32 GBof memory, and an Intel i7 processor CPU with 3.9 GHz.

The Adam optimization algorithm, a special implementation of stochastic gradient descent, is described in detail in Reference [41]. In line with other fine-tuning applications, the learning rate was set as $10^{-5}$, and the loss function used during training is given by

(2) $$L\;=\;-\frac{1}{m}\overset{m}{\underset{i=1}{\sum }}\overset{n}{\underset{j=1}{\sum }}b\left(i,j\right)\log_{10}\left(p_{i,j}\right),$$

where

(3) $$b\left(i,j\right)\;=\;\left\{\begin{array}{cc} 1, & y^{\left(i\right)}\;=\;j,\\ 0, & \mathrm{otherwise}. \end{array}\right.$$

This calculates the loss for each observation *i* for each label *j* for a number of categories n ≥ 2, and $p_{i,j}$ is the model’s predicted probability for the label *j* of the *i*th input.

#### 2.2.4. Inspection

The CNN provides an output that is representative of its confidence that there is a crack in an image. As previously mentioned, this output is valued between 0 and 1. The lower extreme value (0) indicates that there is no crack in an input image, and the upper extreme value (1) means that an input image contains a crack. The output value between the two values represents the percentage of confidence. It is further interpreted via the crack detection algorithm by an introduction of the user-defined thresholds $\gamma_{c}$ and $\gamma_{\mathrm{nc}}$ that determine the algorithm’s classification of the image. If the output is below $\gamma_{\mathrm{nc}}$, it is determined that there are no cracks in the image. The judgement for the image is that there is a crack if the output value is larger than $\gamma_{c}$. If the output lies between $\gamma_{\mathrm{nc}}$ and $\gamma_{c}$, the image is classified as uncertain. For instance, if one sets $\gamma_{c}$ and $\gamma_{\mathrm{nc}}$ as a high value (i.e., 0.95 or more) and a low value (i.e., 0.05 or less), respectively, it can be said that the user wants to determine the presence of a crack only for an image that has definite confidence (95%) by applying strict conditions. In addition, pixel information for the center of the input image is determined for images with positive identification for cracks, and this information is included in the output of the algorithm.

The image to be inspected typically has a higher resolution than what may be placed into the VGG16 model, which is designed for a 224 by 224 pixel Red-Green-Blue image. Inputting the higher resolution image to the CNN can require significant downsampling, which incurs the loss of information. Because of this, the image is divided into sections that are individually inputted into the CNN. In this work, two methods of dividing the image were studied. *The first method (Method 1) divided the image once into equal-sized sections equal to the VGG16’s input size to avoid loss of information incurred by downsampling the larger image to the model’s input size*. If there are sections of the image (called sub-images) where the algorithm’s judgement is uncertain, these subimages are saved to be used in the retraining algorithm. This approach mainly focuses on the accuracy of crack detection. *In the second method (Method 2), the image is initially divided into a number of sections desired by the user, and it iteratively reinspects sections of the image where the algorithm’s judgement is uncertain*. Method 2 primarily focuses on the aspect of a computational burden.

#### 2.2.5. Retraining and Reinspection for Each Method

To minimize uncertainty in the system, Method 1 and Method 2 consider retraining and reinspection processes, respectively. In Method 1, images that were labeled during inspection in the past may be used to retrain the CNN so that the algorithm has increasing performance with continued application of the system. After the inspection, newly labeled data for crack and no-crack images are available for training. Adding these images to the training set will increase its size. The model that was fine-tuned from the ImageNet weights may then be fine-tuned again using the training set augmented with new images, and the retrained model is used to inspect the uncertain images from the initial inspection. After the initial inspection, there are generally more no-crack images than crack images. This is typical because damaged parts of a structure are usually much smaller than the overall size of the structure, so the labeled data added to the training data set can become unbalanced if left alone. For this reason, images from the overrepresented classification are randomly selected to become training data so that the number of crack images and no-crack images remain the same. This helps to avoid biasing the model during retraining. Using a balanced new training data set, retraining is performed in iterations. The remaining uncertain images are inspected and potentially classified as crack or no crack. If a judgement is made, then the newly classified image can be added to the training data set for the next retraining iteration. If it is not possible to balance the data being added to the training data set, however, the retraining stops at that iteration. Although it is a small detail on this work’s application, this would have a great impact on the results when inspecting much larger images.

In Method 2, the size of divided sections will be greater than the input size of the model but closer to the actual size than the original image size. If the algorithm determines a section as uncertain, the image is reinspected by dividing it again, and each subsection can be put into the CNN. Subsections can be reinspected as well, so the algorithm iteratively breaks up the image and reinspects it until the uncertainty is minimized. The algorithm, however, will not continue to divide an image if the sizes of the resulting sections are equal to or less than the VGG16’s input size.

### 2.3. Location Determination

**Figure 3:**
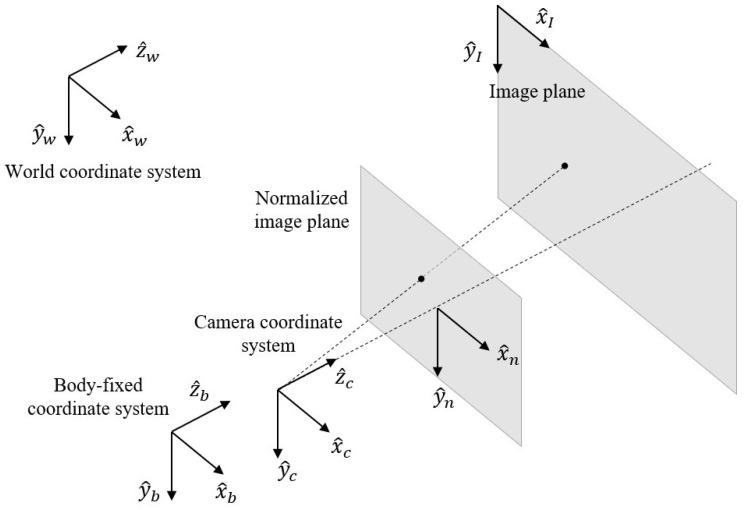
Coordinate systems and image planes.

Through the crack detection system proposed, the confidence in the presence of a crack is returned and pixel information for the center of the divided image including cracks is obtained in order to find the location of cracks in the real world. For this process, three coordinate systems and two image planes are considered in this work, as shown in Figure 3. When taking a photo using a camera, an object of interest in the world coordinate system is projected onto the image plane, and there is a mathematical model to describe how points in the world coordinate system become projected onto the image plane. Using the inverse relation of this mathematical model, the real locations of points in the image plane are determined.

First, the relationship between the camera coordinate system and the image plane is described using camera parameters, which are called the intrinsic parameters, such as the focal length, $f_{x}$ and $f_{y}$; the principal point, $c_{x}$ and $c_{y}$; the skew coefficient, $\alpha_{c}$; and *i*th coefficients of the radial and tangential distortion coefficients, $k_{r,l}$ and $k_{t,m}$, where l = 1, 2, 3 and m = 1, 2. These intrinsic parameters are estimated by using the Camera Calibration Toolbox [42]. The projection from the camera coordinate system onto the image plane is described in a general mathematical model using the intrinsic parameters as follows: [42,43]

(4) $$\left(\begin{array}{c} x_{d}\\ y_{d}\\ 1 \end{array}\right)\;=\;\left(\begin{array}{c} \left(1\;+\;r^{2}k_{r,1}\;+\;r^{4}k_{r,2}\;+\;r^{6}k_{r,3}\right)x_{n}\;+\;2x_{n}y_{n}k_{t,1}\;+\;k_{t,2}\left(r^{2}\;+\;2x_{c}^{2}\right)\\ \left(1\;+\;r^{2}k_{r,1}\;+\;r^{4}k_{r,2}\;+\;r^{6}k_{r,3}\right)y_{n}\;+\;k_{t,1}\left(r^{2}\;+\;2y_{c}^{2}\right)\;+\;2x_{n}y_{n}k_{t,2}\\ 1 \end{array}\right),$$

where ${\left(x_{n},\;y_{n},\;1\right)}^{T}\;=\;{\left(x_{c}/z_{c},\;y_{c}/z_{c},\;1\right)}^{T}$, $z_{c}$ is the distance between the camera and the subject and $r^{2}\;=\;x_{n}^{2}\;+\;y_{n}^{2}$. Once distortions are applied, the point in the image plane ${\left(x_{i}\;y_{i}\right)}^{T}$ is derived as [42,43]

(5) $$\left(\begin{array}{c} x_{i}\\ y_{i}\\ 1 \end{array}\right)\;=\;K\left(\begin{array}{c} x_{d}\\ y_{d}\\ 1 \end{array}\right),$$

where

(6) $$K\;=\;\left[\begin{array}{ccc} f_{x} & \alpha_{c}f_{x} & c_{x}\\ 0 & f_{y} & c_{y}\\ 0 & 0 & 1 \end{array}\right].$$

Equations (4) and (5) express the projection from the camera coordinate to the image plane. The points in the camera coordinate system are calculated using the inverse process mentioned if the points in the image plane are given. However, there is a nonlinear calculation to calculate inversely in Equation (4). If images that are compensated using distortion coefficients, then ${\left(x_{n},\;y_{n},\;1\right)}^{T}\;=\;{\left(x_{d},\;y_{d},\;1\right)}^{T}$. Thus, this leads to $p_{c}\;=\;{\left(x_{c},\;y_{c},\;z_{c}\right)}^{T}\;=\;z_{c}K^{-1}\;{\left(x_{i},\;y_{i},\;1\right)}^{T}$.

To find the location of the point in the world coordinate system, the rotation matrix, $R_{CW}$, which maps the world coordinate system into the camera coordinate system, is used. Then, the location of the point in the world coordinate system, $p_{w}\;=\;{\left(x_{w},\;y_{w},\;z_{w}\right)}^{T}$, is provided as [42]

(7) $$p_{c}\;+\;p_{w}\;=\;R_{CW}^{T}r_{t}^{T},$$

where $r_{t}\;=\;{\left(r_{x},\;r_{y},\;r_{z}\right)}^{T}$ is the translation vector from the origin of the world coordinate system to the camera coordinate system. If the images are taken by a UAV, the position and attitude information of the UAV can be obtained. Note that the relationship between the UAV and the camera is easily calculated because the camera is fixed in the UAV. $R_{CW}$ is provided from the attitude of the UAV, and $r_{t}$ is obtained from the position data of the UAV. It is assumed that the camera coordinate system does not rotate with respect to the world coordinate system because images were taken using a quadcopter in hovering mode in this work.

## 3. Experiment Results

### 3.1. Experiment Conditions and Preparations

To validate the proposed system, experiments were carried out. Images directly taken by humans have very good conditions for detecting cracks and image processing, but it is possible for structural cracks to be located in unreachable places by inspectors without the proper equipment. For this reason, images taken by a UAV are considered in this work because it is possible to acquire visual information for large structures such as bridges or tall buildings. In this work, two images taken by the UAV in hovering mode were utilized by the system proposed. Note that the UAV is controlled such that the optical axis (${\hat{z}}_{c}$) of the camera is perpendicular to the wall. Then, it reduces the effect of camera lenses’ distortion. Photos of cracks on the side of a wall were taken at the Art Gallery at Mississippi State University with the camera installed in the drone: 12 mega-pixel sensor and f/2.2 aperture.

### 3.2. Feature-Based Image Processing

**Figure 4:**
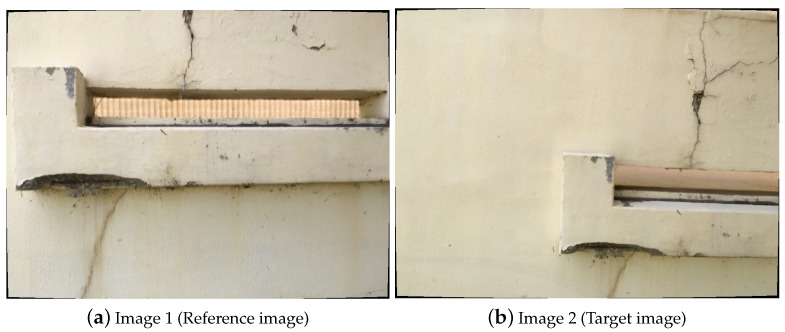
Undistorted images for which the distortions will be corrected.

Before performing the process, estimating the camera parameters is needed because the intrinsic parameters of the camera are utilized to determine the location of points on the images. Additionally, the distortion coefficients in the intrinsic parameters are applied to the images taken by the UAV in order to compensate for distortion made by lenses, and the un-distorted images shown in Figure 4 are utilized for the image stitching process to make a large image map. In between two images, Image 1 is the reference image in the image processing step and Image 2 is stitched on Image 1 based on the common features obtained from the two images.

**Figure 5:**
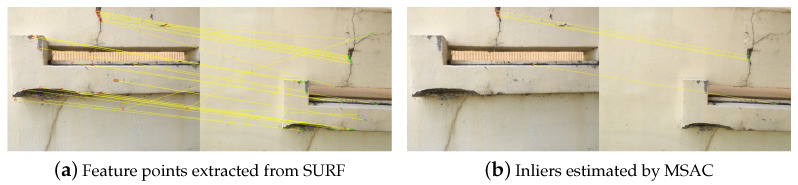
Results for applying Speeded Up Robust Features (SURF) and M-estimator Sample Consensus (MSAC).

The black background at each corner of the undistorted images represents the distortion, so only the valid rectangular part of these images is used to apply the image stitching algorithm. First, the common feature points in these two images are extracted using SURF, as shown in Figure 5a. A number of features including outliers are shown in the result figures. Based on these features, the correct matching sets, the inliers, are estimated from MSAC, as shown in Figure 5b, and the geometric relationship is calculated using these inliers. The angle and scale of Image 2, which should be transformed, with respect to Image 1 are obtained as the angle of 3.6836° and scale of 1.1622, respectively. Although the UAV was in hovering mode in the experiment, the angle and scale between the two images are different because of disturbances, such as wind.

**Figure 6:**
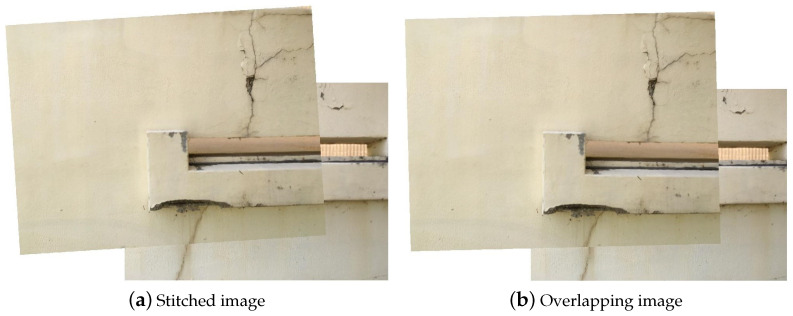
Results for the stitched and overlapping images.

After the geometric relationship is applied to the images, the stitched image is obtained as shown in Figure 6a. Some differences between the two images are found because the attitude of the UAV is somewhat affected by control errors and/or disturbances. If sufficient inliers are not found by MSAC, stitching images are not available since the geometric information cannot be estimated due to a lack of information. In this case, overlapping images based on the UAV information should be considered as shown in Figure 6b. Two images are not well-overlapped because of the measurement error. This mismatched part may cause false identification. For this reason, it is better to use the stitched image for crack detection if possible.

### 3.3. Crack Detection and Location Determination

To inspect the images for cracks, the model must first be trained. With the trained crack detector, the stitched image is processed by two methods. Method 1 breaks the image into equal-sized sections with 224 by 224 pixel resolution that is the same as the required input size of the CNN in order to prevent loss of information and retrains on some of the newly labeled images to minimize uncertainty in the judgement. Method 2 breaks the stitched image into a number of sections chosen by the user and reinspects these images to minimize uncertainty in the judgement.

It should be noted that, in the image, there is damage to the structure that a human may not clearly identify as a crack. This damage can be seen on the bottom-left corner of the protruding structure in Figure 6a. Certainly, the structure there is not intact, but its classification as a crack is incorrect. In this work, *the features of cracks are considered long and slender damages in concrete texture with a dark interior relative to the surrounding surface representing its depth into the structure*. These features are well-represented in the data set and visualized in the following subsections.

#### 3.3.1. Initial Model Training

**Figure 7:**
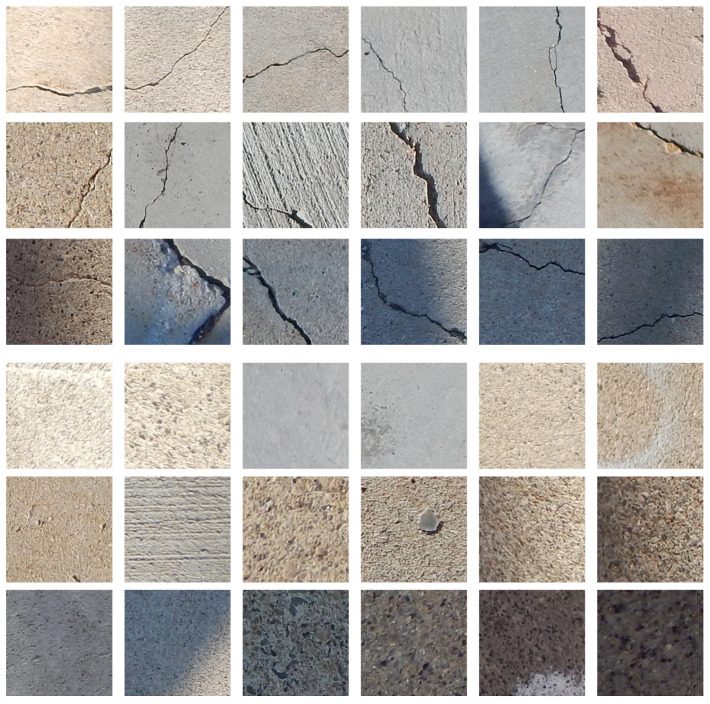
Representation of the images used to train labelling of crack images (the first three rows) and no-crack images (the last three rows).

**Figure 8:**
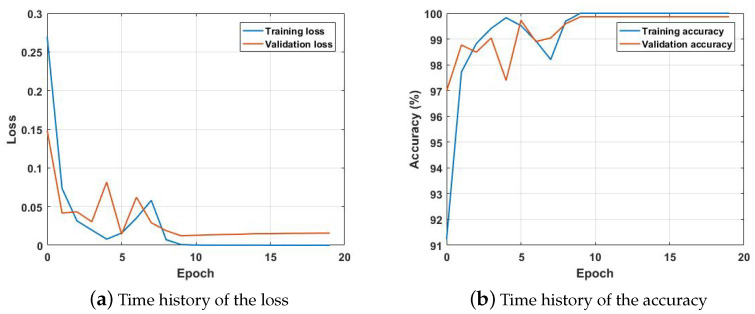
Time history of the loss and accuracy for the initial training.

Before inspection of the stitched image, a model was trained using the 3600 hand-picked images from the SDNet data set. The model was trained in batches of 36 images, so in every step, 36 images were inspected by the model and used to calculate loss and gradient for the optimization algorithm. Examples of the images used in the crack and no-crack categories are shown in Figure 7. Since image acquisition is generally performed during the daytime, the images taken in a normal daylight condition are selected as a training data set. There were 80 steps per epoch of training and 20 steps per epoch of validation. Training continued until either the validation accuracy stopped improving or the training reached 100 epochs. Each epoch required only 26 s to be completed, averaging 326 milliseconds per step. The history for the training is shown in Figure 8. Figure 8a is the result for the loss calculated using Equation (2), and Figure 8b shows the accuracy of the model during training and validation, which is the percentage of images classified correctly during training and validation. During both validation and training, the accuracy is well above 99%.

#### 3.3.2. Method 1

**Figure 9:**
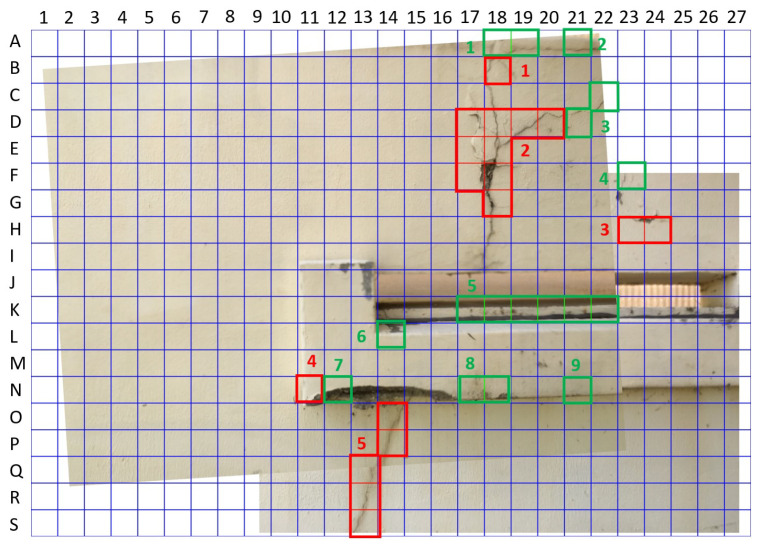
Initial result of crack detection using Method 1.

**Figure 10:**
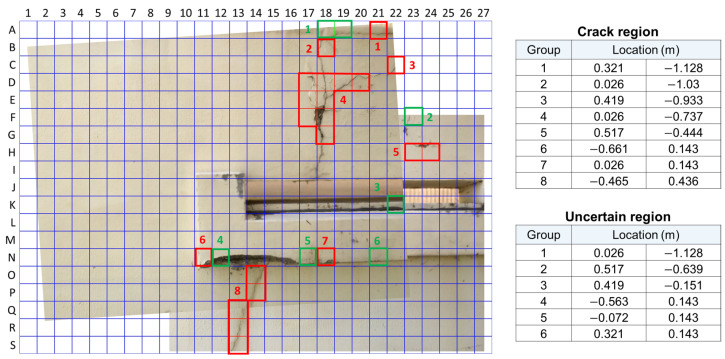
Final result of Method 1 for human inspectors.

The stitched image was broken up into 513 sections, divided 27 times along the *x*-axis and 19 times along the *y*-axis, with 224 by 224 pixel resolution. The thresholds for identifying cracks have a large uncertain interval as $\gamma_{c}\;=\;0.9$ and $\gamma_{nc}\;=\;0.1$ in order to reduce misdetections arising from uncertain information. Initially, 18 red-boxed sections were identified as containing cracks and 17 green-boxed sections were determined as uncertain, as shown in Figure 9. However, through retraining, the total number of uncertain images was reduced to just 7, as shown in the left picture of Figure 10. Additionally, the number of uncertain regions is reduced from 9 to 6. Retraining took only two iterations in this scenario because it could not balance the data set for training in the next iteration.

In order to reduce the burden on inspectors to view many images output by the system, some sections can be amalgamated into local regions, grouped by their proximity to one another. Figure 9 and Figure 10 contain the results of grouping the labeled sections into disparate regions. This result provides inspectors with a clearer picture of the damage found by the crack detector compared to the cluttered result depicting the result for every section.

**Figure 11:**
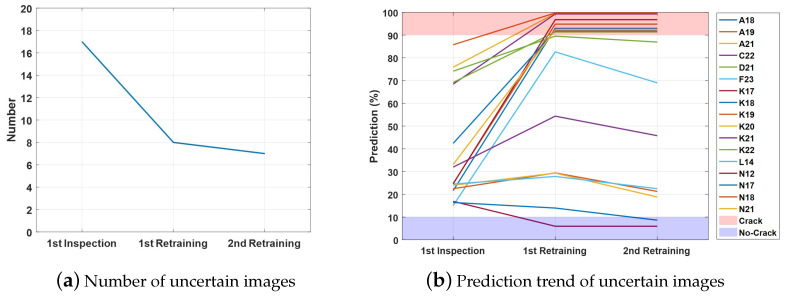
Number of uncertain images and its prediction trend.

Figure 11a shows the number of uncertain images dropping with the increasing number of iterations. As shown in Figure 11b, between the first and second iterations, the judgement for section K18, which can be found in Figure 9 and Figure 10, changed from uncertain to no crack. However, there were no newly classified crack images available to make the new training data set balanced for the next iteration, so retraining stopped at the second iteration.

**Figure 12:**
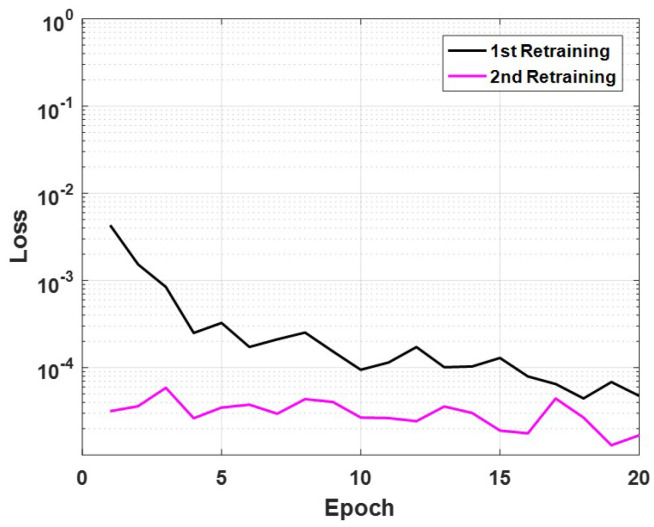
Time history of the loss for retraining the model in Method 1.

The value of the loss function during each retraining step is shown in Figure 12. The plots of these values demonstrate that retraining increases the model’s performance when given augmented data, which are 18 crack and 18 no-crack images. The reduction of loss in the first step was small, only on an order of magnitude of $10^{-3}$, but it was enough to change the judgement of 10 sections. The change in the second step was even smaller, but again, it changed the judgement of a section.

Pixel information for the center points of the divided images as well as the confidence in the presence of a crack are obtained from the crack detection procedure. This pixel information is mapped from the image plane into the world coordinate system using intrinsic parameters, and the distance between the structure and the camera is easily obtained since the distortions are already compensated in the stitched image. The distance measured by the range sensor is about 1.5 m for Image 1 in this experiment. The rotation matrix is assumed as the identity matrix because the coordinate systems are not rotating. Additionally, the translation vector given by the position information of the UAV is assumed as a zero vector, which means that the origin of the camera coordinate system is placed in the origin of the world coordinate system. The outputs of the crack detection and the determined location information are listed on the right side of Figure 10. In these results, the direction of *y*, which is downward, should be noted because the direction is opposite to the general direction. The location for each region is calculated using an average value, but if the calculated location is not placed on that region, the nearest location among images from the calculated location is set as the region’s location. The whole results for each subsection about the crack and uncertain images are listed in Table 1. The region map and its location information are provided for human inspectors as a report shown in Figure 10. The classified region map and its locations were gathered in one scene for ease of understandability. In the case of the images of uncertain regions, inspection by human inspectors is needed to classify whether there is a crack because the proposed system was not able to detect cracks. After this process, the judged image are labeled as crack or no crack for the retraining process for future inspections.

#### 3.3.3. Method 2

**Figure 13:**
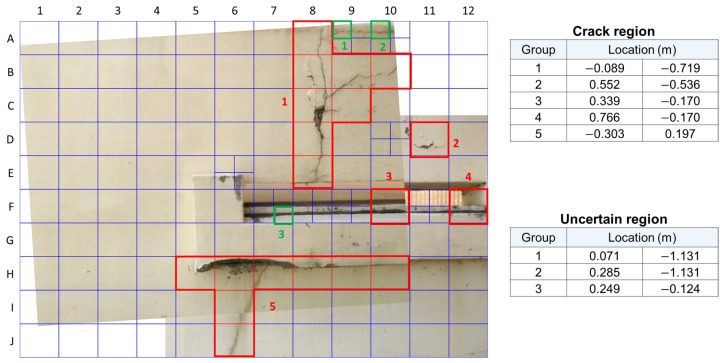
Final result of Method 2 for human inspectors.

The user defines the number of times that the image is divided. The image was divided into 120 sections, 12 divisions along the *x*-axis and 10 divisions along the *y*-axis. Furthermore, if the judgement of a section is uncertain, it is divided into 4 sections of equal size to be reinspected. In this way, uncertainty in the model’s judgement of the image’s sections is reduced through reinspection. There were very few uncertain sections as a result of this method, so retraining was not utilized in Method 2. The outputs after reinspection and amalgamated location information are shown in Figure 13, and all location information is listed in Table 2.

Many sections of the protruding structure that do not have cracks are identified as having cracks. Much of the damage to the bottom-left section of the protruding structure was judged as no crack in Method 1. This may have to do with the relative size of the damage in the different sizes of divisions. The greater the size of the section, the more slender and, thus, more crack-like the damage can look. The CNN can only use the features present in the image to make its judgement, and the relative size and slenderness of the damage can have a significant effect. This is likely to be the case for the dark edges of the protruding structure as well. At the resolution of these sections, their relative size and slenderness appear more crack-like to the model.

### 3.4. Discussion

Two methods were used to detect cracks with minimal uncertainty. Method 1 has a computationally intensive process because it contains the retraining process, but it improves the accuracy. On the other hand, the number of misidentifications of Method 2 is relatively larger than that of Method 1, but the computational time is negligibly small because Method 2 only reinspects uncertain images without the retraining process. Although both methods were proposed to provide situation-dependent flexibility to users, the authors believe that Method 1 will be more practical in the real world, which takes into account accuracy as a more critical factor than the computational burden. For interested readers, another test result using Method 1 for a larger structure is included in Appendix A.

## 4. Conclusions

In this work, a framework was proposed to detect cracks that are defined as long and slender damages with dark interiors with concrete texture in a large image using a deep learning model and to determine the cracks’ locations on the structure. To validate the proposed system, experiments were carried out using images taken of a real structure by a UAV. The obtained images were stitched into one large image to overcome the limitation of the resolution of the camera. Next, a CNN was used to detect any cracks in the image that was divided into several sections to label cracks in several regions of the image. To minimize uncertainties in crack detection, this work proposed two methods considering retraining and reinspection processes. With the output of the crack detection process and UAV sensors’ information, cracks’ locations in the world coordinate system were provided. Finally, the proposed system created a cohesive map of the inspected area and outlined regions of cracks and uncertainty. In other words, for large structures, the proposed framework can provide an entire map with highlighted regions of cracks and uncertain parts and their location information in the world coordinate system. This crack region map helps inspectors improve their ability to inspect large structures. Additionally, in between the two approaches proposed, Method 1 providing accurate outcomes will be a good option for crack detection because postprocessing is generally utilized to detect cracks. As a follow-up project, further research will be conducted in order to improve the current framework by including the length and width quantifications and by considering various types of cracks in the training data set and to provide the time history of the crack spread.

## Figures and Tables

**Table 1:** Results of the crack detection and its location for Method 1.

ID	Classification	Prediction (%)	Pixel		Location (m)	
A21	crack	92.75	4592	112	0.321	−1.128
B18	crack	98.13	3920	336	0.026	−1.030
C22	crack	93.48	4816	560	0.419	−0.933
D17	crack	99.99	3696	784	−0.072	−0.835
D18	crack	99.96	3920	784	0.026	−0.835
D19	crack	99.99	4144	784	0.125	−0.835
D20	crack	99.99	4368	784	0.223	−0.835
E17	crack	99.62	3696	1008	−0.072	−0.737
E18	crack	99.99	3920	1008	0.026	−0.737
F17	crack	99.94	3696	1232	−0.072	−0.639
F18	crack	99.99	3920	1232	0.026	−0.639
F18	crack	99.99	3920	1456	0.026	−0.542
H23	crack	97.28	5040	1680	0.517	−0.444
H24	crack	99.91	5264	1680	0.616	−0.444
N11	crack	96.34	2352	3024	−0.661	0.143
N18	crack	96.86	3920	3024	0.026	0.143
O14	crack	94.29	3024	3248	−0.367	0.240
P14	crack	90.21	3024	3472	−0.367	0.338
Q13	crack	99.90	2800	3696	−0.465	0.436
R13	crack	99.99	2800	3920	−0.465	0.534
S13	crack	90.96	2800	4144	−0.465	0.631
A18	uncertain	77.65	3920	112	0.026	−1.128
A19	uncertain	37.61	4144	112	0.125	−1.128
F23	uncertain	17.71	5040	1232	0.517	−0.639
K22	uncertain	20.31	4816	2352	0.419	−0.151
N12	uncertain	25.37	2576	3024	−0.563	0.143
N17	uncertain	13.37	3696	3024	−0.072	0.143
N21	uncertain	65.04	4592	3024	0.321	0.143

**Table 2:** Results of the crack detection and its location for Method 2.

ID	Classification	Prediction (%)	Pixel		Location (m)	
A8	crack	99.98	3656.87	209.85	−0.089	−1.085
B8	crack	99.99	3656.87	629.55	−0.089	−0.902
B9	crack	99.99	4144.46	629.55	0.125	−0.902
B10	crack	99.68	4632.04	629.55	0.339	−0.902
C8	crack	99.99	3656.87	1049.25	−0.089	−0.719
C9	crack	98.92	4144.46	1049.25	0.125	−0.719
D8	crack	99.99	3656.87	1468.95	−0.089	−0.536
D11	crack	99.99	5119.63	1468.95	0.552	−0.536
E8	crack	99.82	3656.87	1888.65	−0.089	−0.353
F10	crack	91.61	4632.04	2308.35	0.339	−0.170
G0	crack	92.85	5607.21	2308.35	0.766	−0.170
H5	crack	99.83	2194.13	3147.75	−0.731	0.197
H6	crack	99.22	2681.71	3147.75	−0.517	0.197
H7	crack	99.99	3169.29	3147.75	−0.303	0.197
H8	crack	99.99	3656.87	3147.75	−0.089	0.197
H9	crack	99.60	4144.46	3147.75	0.125	0.197
H10	crack	94.07	4632.04	3147.75	0.339	0.197
I6	crack	96.44	2681.71	3567.45	−0.517	0.380
J6	crack	99.99	2681.71	3987.15	−0.517	0.563
A9-1	uncertain	14.81	4022.46	105.10	0.071	−1.131
A10-1	uncertain	62.64	4510.29	105.10	0.285	−1.131
F7-4	uncertain	13.96	3291.29	2413.35	−0.249	−0.124

**Figure A1:**
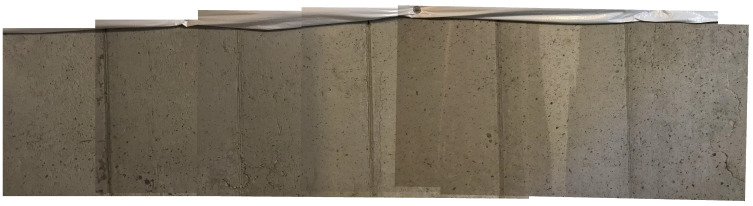
Stitched image.

**Figure A2:**
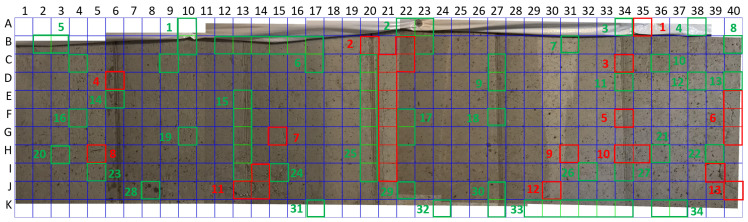
Initial result of crack detection using Method 1.

**Figure A3:**
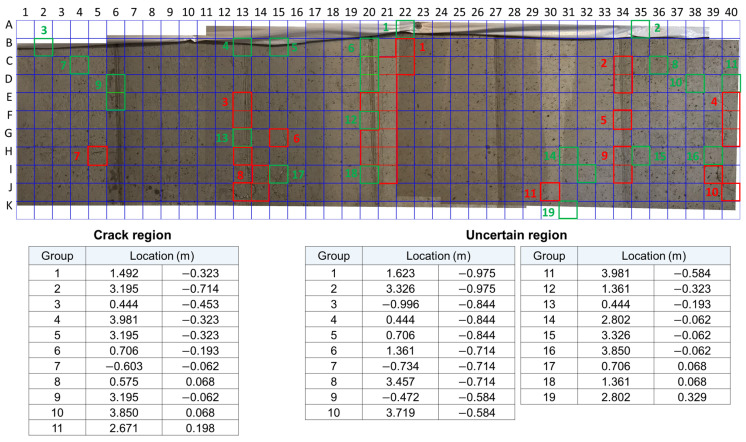
Final result of Method 1 for human inspectors.

## Appendix A

A total of 6 images are obtained to cover a 5 m × 1.5 m region, and the stitched image is depicted in Figure A1. The stitched image was broken up into 440 sections, divided 40 times along the *x*-axis and 11 times along the *y*-axis, with 224 by 224 pixel resolution. The thresholds for identifying cracks and no cracks are set as $\gamma_{c}\;=\;0.94$ and $\gamma_{nc}\;=\;0.18$ for this experiment. Again, these thresholds can be defined by users to determine the algorithm’s classification of the image. Figure A2 shows the initial results of crack detection using Method 1, and Figure A3 contains the results of grouping the labeled sections into crack and uncertain regions as well as the amalgamated location information from H9, which is the origin of the reference coordinate.
